# Genetic Models of Rare Diseases: A Call for Papers

**DOI:** 10.1093/g3journal/jkac189

**Published:** 2022-08-11

The journals of the Genetics Society of America, GENETICS and G3: Genes|Genomes|Genetics, are calling for submissions of papers in the area of Genetic Models of Rare Diseases.

Identifying genes associated with rare diseases, and understanding how variants within those genes alter molecular and cellular phenotypes, is a central challenge. The advent of whole exome sequencing (WES) and whole genome sequencing (WGS) has dramatically changed the landscape of human genetics. Sequencing can be used to identify variants in genes that may be disease causing, driving the discovery of rare genetic diseases. These approaches can pinpoint a gene or variant of interest based on the sequence from a few family members rather than extensive family histories. However, the genes and variants of interest often benefit from modeling in cellular assays or genetic model organisms to understand molecular and cellular mechanisms of disfunction. Model organisms have therefore been useful for the discovery of new genetic diseases, and are key to understanding variant effects. It is estimated that the genetic variants for thousands of rare diseases remain to be discovered. Hence, much work lies ahead.

A key advantage to modeling a disease gene in genetic model organisms is that the gene or variant function can be explored in depth. For example, molecular effects on metabolome, transcription, translation or protein function can be assayed, or transcriptomes or interactomes can be profiled. These studies can pinpoint disease mechanism, reveal unanticipated gene functions or elucidate specific existing pathways. These discoveries can identify other genes that may cause similar phenotypes, driving the discovery of additional, related rare human diseases. High-throughput assays such as Multiplexed Assays of Variant Effect (MAVEs) have also opened the door to systematic assessment of the functional consequences of genetic variation in human disease genes. Unraveling the mechanism by which variants act can highlight potential therapeutic strategies to help patients. Hence, organisms like yeast, worms, flies, fish and mice (as well as other genetic models) can be particularly useful.

This series will highlight ongoing advances in rare disease discovery and mechanisms by presenting key research findings, new discoveries, and reviews or perspectives. We invite high-quality submissions with a focus on model organism-human genetics, genomics, MAVE studies and work that leverages advanced genomic tools for gene identification and editing to address the issues noted above.

**Series Editors:**

- Brenda Andrews (University of Toronto)
- Hugo Bellen (Baylor College of Medicine)
- Douglas Fowler (University of Washington School of Medicine)
- Philip Hieter (University of British Columbia)

The Genetic Models of Rare Diseases Series will launch in Summer 2023; though articles will be published online shortly after acceptance via Advanced Access (including the completed proof). Papers will be collected and navigable across the two journals and promoted on social media and other venues, in a manner similar to other series the journals have published.

Authors are invited to **submit manuscripts by 16 January 2023** and should submit to the journal of choice. Manuscripts will be reviewed and edited through the standard peer review process and according to the usual high standards of the journals. Some manuscripts submitted to GENETICS may be offered a transfer to G3: Genes|Genomes|Genetics instead.

We encourage continued submissions past 16 January 2023 as we intend this series to be an ongoing and growing resource in this area. At submission, please choose the “Genetic Models of Rare Diseases” article type in the submission systems and mention the series in your cover letter.

Please share with your colleagues, and do not hesitate to contact any of the series editors or the GENETICS (genetics-gsa@thegsajournals.org) and G3: Genes|Genomes|Genetics (g3-gsa@thegsajournals.org) Editorial Office with questions, suggestions, or presubmission inquiries.

